# Targeting *EGFR* exon 20 insertion mutations in non-small cell lung cancer

**DOI:** 10.1038/s41392-019-0038-9

**Published:** 2019-03-08

**Authors:** Simon Vyse, Paul H. Huang

**Affiliations:** Division of Molecular Pathology, The Institute of Cancer Research, London, SW3 6JB United Kingdom

**Keywords:** Lung cancer, Biochemistry, Molecular medicine

## Abstract

Inframe insertions of three or more base pairs in exon 20 of the epidermal growth factor receptor (*EGFR)* gene were among the first *EGFR* mutations to be identified as oncogenic drivers in non-small cell lung cancer (NSCLC). However, unlike the classical *EGFR* L858R point mutation or exon 19 deletions, which represent the majority of *EGFR* mutations in NSCLC, low frequency *EGFR* exon 20 insertion mutations are associated with de novo resistance to targeted EGFR inhibitors and correlate with a poor patient prognosis. Here, we review the developments over the last 5 years in which pre-clinical studies, including elucidation of the crystal structure of an EGFR exon 20 insertion mutant kinase, have revealed a unique mechanism of kinase activation and steric conformation that define the lack of response of these *EGFR* mutations to clinically approved EGFR inhibitors. The recent development of several novel small molecule compounds that selectively inhibit *EGFR* exon 20 insertions holds promise for future therapeutic options that will be effective for patients with this molecular subtype of NSCLC.

## Tailoring therapies for lung cancer

Activating mutations in the epidermal growth factor receptor (*EGFR*) gene are commonly observed in non-small-cell lung cancer (NSCLC). Treatment with EGFR inhibitors has proven successful in many patients harboring such mutations, but patients with EGFR exon 20 insertion mutations do not respond these drugs. Simon Vyse and Paul H Huang at The Institute of Cancer Research in London, UK, review the latest research on the effect that the insertion of three or more nucleotide base pairs into EGFR exon 20 has on the receptor’s structure and activity, which contributes to its poor sensitivity to currently available EGFR inhibitors. Promising preclinical findings with compounds that selectively target the EGFR exon 20 insertion mutant protein may lead to new treatment options for patients with this form of NSCLC.

## Introduction

Lung cancer is the most common malignant disease and the leading cause of cancer mortality worldwide, with non-small cell lung cancer (NSCLC) comprising the vast majority (85%) of all lung malignancies.^1,2^ Over the last decade, our understanding of NSCLC has evolved beyond broad histological subtypes and ‘one size fits all’ treatment approaches toward a refined disease classification underpinned by defined genetic alterations and precision therapies guided by molecular stratification. For the adenocarcinoma subtype of NSCLC in particular, significant improvements in progression-free survival (PFS) have been achieved for patients with epidermal growth factor receptor (*EGFR*) mutations, anaplastic lymphoma kinase (*ALK*) translocations, ROS1 proto-oncogene receptor tyrosine kinase (*ROS1*) rearrangements and, B-raf proto-oncogene, serine/threonine kinase (*BRAF*) mutations due to the effectiveness and availability of therapies that specifically target these molecular drivers.^3–7^ Among these advances, the successful use of EGFR inhibitors to treat *EGFR* mutant positive adenocarcinoma and the subsequent development of third generation inhibitors to tackle acquired drug resistance have become the poster child of targeted therapy in oncology.^8,9^

A series of studies in the early 2000s observed that distinct epidemiological subgroups of patients with NSCLC (correlating with women, Asian populations and non-smokers) had dramatically enhanced responses to treatment with the ATP-competitive, reversible EGFR inhibitors gefitinib and erlotinib.^10–12^ In multiple reports, the vast majority of gefitinib-responsive or erlotinib-responsive lung cancers were found to harbor somatic *EGFR* mutations, while no *EGFR* mutations could be detected in non-responsive patients.^13–15^ These pioneering studies indicated that the presence of *EGFR* mutations predicted increased sensitivity to EGFR inhibitors, a finding that has subsequently been supported by numerous follow-up studies. The Iressa Survival Evaluation in Lung Cancer (ISEL) clinical trial, for example, compared gefitinib treatment to placebo in NSCLC and found that the response rate (RR) to gefitinib was much higher (37.5%) in patients who possessed *EGFR* mutations compared to those without (2.6%).^16^ Another study, the Iressa Pan-Asia Study (IPASS) trial, compared gefitinib treatment to carboplatin plus paclitaxel and showed an extraordinary 72.1% versus 1.1% RR to gefitinib for patients with and without *EGFR* mutations, respectively, alongside significantly increased PFS in the gefitinib-treated, *EGFR* mutation-positive group.^17^ Subsequently, the phase III European Tarceva vs. Chemotherapy (EURTAC) trial also showed a significant advantage for erlotinib (64% RR, 9.7 months PFS) in *EGFR* mutant patients compared to cisplatin or carboplatin plus either docetaxel or gemcitabine chemotherapy (18% RR, 5.2 months PFS).^4^

**Fig. 1:**
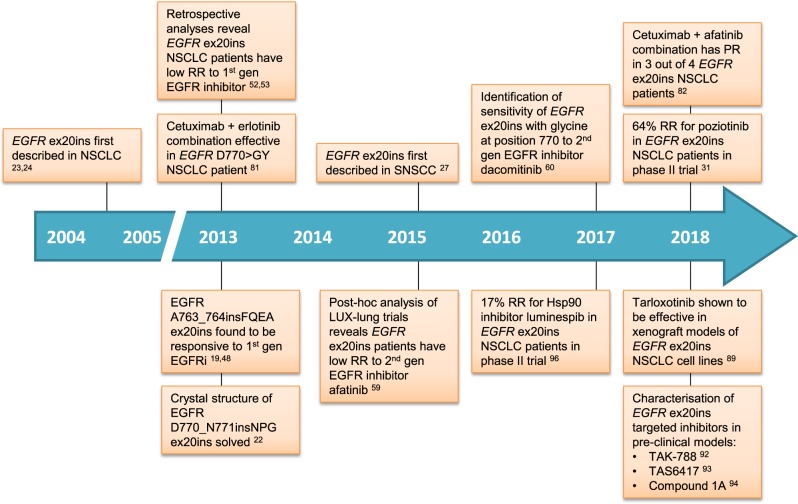
Developments in understanding *EGFR* exon 20 insertion-positive NSCLC. A timeline of key clinical and pre-clinical studies that have established the response of *EGFR* exon 20 insertion NSCLC to EGFR inhibitors and recent progress towards the development of novel therapeutic strategies for this molecular subtype

The single point mutation leucine-858 to arginine (L858R) in exon 21 and variable deletions of at least three amino acid residues in exon 19 are together often referred to as ‘classical’ *EGFR* activating mutations and represent the vast majority (85–90%) of all observed *EGFR* kinase domain mutations in NSCLC.^18^ It is now clear, however, that not all activating *EGFR* mutations are inherently sensitive to EGFR inhibitors. Inframe base pair insertions in exon 20 also result in constitutive activation of EGFR, but unlike the classical activating *EGFR* mutations, *EGFR* exon 20 insertions are associated with de novo resistance to current clinically available EGFR inhibitors.^19,20^ Low response rates of between 3–8% for erlotinib, gefitinib and the second generation EGFR inhibitor afatinib have been reported in *EGFR* exon 20 insertion mutant NSCLC patients (Table 1),^21,22^ and thus, effective treatment options are limited. In recent years, pre-clinical work has sought to establish the reasons underlying the failure of EGFR inhibitors in NSCLC patients harboring *EGFR* exon 20 insertion mutations. Several candidate inhibitors have since been developed that selectively target the *EGFR* exon 20 insertion mutant kinase (Fig. 1). Moving forward, these novel EGFR inhibitors are poised to provide viable therapeutic options that could soon benefit patients with this molecular subtype of NSCLC.

**Table 1:** Key clinical trials in *EGFR* exon 20 insertion positive NSCLC

Inhibitor(s)	Target(s)	Clinical trial ID(s)	Key results	Refs
Gefitinib/Erlotinib	EGFR	Retrospective analysis of clinical studies	<3 months PFS 8–27% RR	^52, 53^
Dacomitinib	EGFR/HER2/HER4	NCT00225121	PR for 1 patient with D770delinsGY	^61^
Afatinib	EGFR/HER2/HER4	NCT00525148 NCT00949650 NCT01121393	8.7% RR, 2.7 months PFS	^59^
Neratinib	EGFR/HER2/HER4	NCT00266877	0% RR	^55^
Osimertinib	EGFR T790M	NCT03414814	Ongoing	^67– 69^
Poziotinib	EGFR/HER2	NCT03066206	Ongoing, 64% RR	^31^
Cetuximab + erlotinib	EGFR	NCT00895362	D770>GY patient with 3.5 years PFS	^81^
Cetuximab + afatinib	EGFR	NCT03727724	Preliminary report, 3 out of 4 ex20ins patients with PR, 5.4 months PFS	^82^
Luminespib	Hsp90	NCT01854034	17% RR, 2.9 months PFS	^96^
Tarloxotinib	EGFR	–	Pre-clinical inhibition of ex20ins EGFR	^89^
TAK-788	EGFR/HER2 ex 20 ins	NCT02716116	Ongoing, preliminary anti-tumor activity reported	^92^
TAS6417	EGFR ex 20 ins	–	Pre-clinical inhibition of ex20ins EGFR	^93^
Compound 1A	EGFR/HER2 ex 20 ins	–	Pre-clinical inhibition of ex20ins EGFR	^94^

Details for trials with NCT numbers can be accessed on https://clinicaltrials.gov/

*PFS* progression-free survival, *PR* partial response, *RR* response rate, *ex20ins* exon 20 insertion

## Incidence of *EGFR* exon 20 insertions in NSCLC

**Fig. 2:**
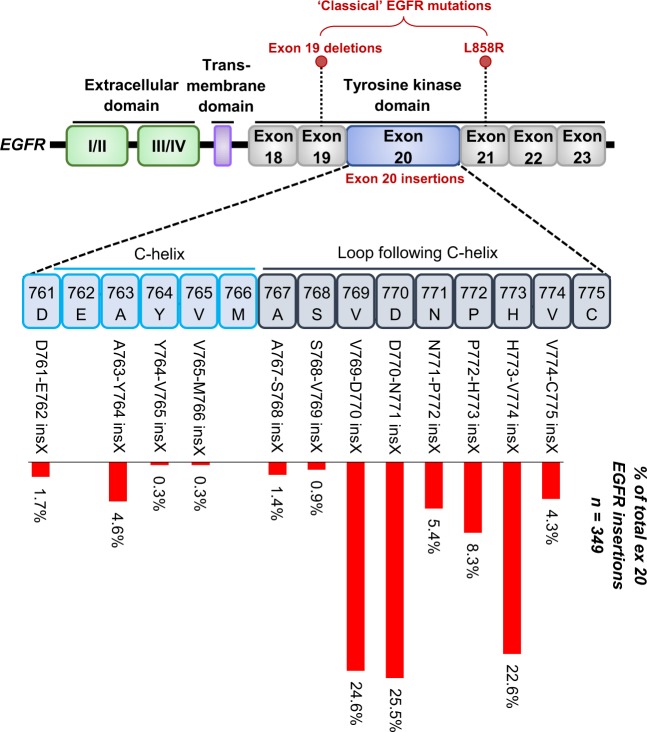
*EGFR* Exon 20 insertion mutations in NSCLC. Within NSCLC, all *EGFR* mutations are clustered across exons 18–22 which encode the tyrosine kinase domain. In particular, 4–10% of *EGFR* mutations are heterogeneous in-frame insertions of between 1–7 amino acids (indicated as ins X) across a span of ~15 amino acids (D761–C775) in exon 20. The prevalence of exon 20 insertions that occur at different amino acid positions are shown by the red bars. Mutations occur within the C-terminal end of the C-helix or more frequently in the loop that immediately follows. Mutation frequency distribution was calculated using COSMIC v86 (http://cancer.sanger.ac.uk) after filtering for NSCLC adenocarcinomas harboring exon 20 insertions (*n* = 349)^98^

*EGFR* exon 20 insertion mutations are heterogeneous at the molecular level but can be characterized as inframe insertions or duplications of between 3 and 21 bp (corresponding to 1 to 7 amino acids) clustered between amino acid positions 762 and 774 of the EGFR protein.^22^ The most common insertion sites in *EGFR* in NSCLC are shown in Fig. 2. Exon 20 insertions were among the earliest *EGFR* mutations identified in NSCLC alongside exon 19 deletions and L858R mutations.^23,24^ However, reports regarding the incidence and clinical outcome of NSCLC patients with these insertions were initially limited. The frequency of *EGFR* exon 20 insertions has since been reported as being between 4 and 10% of all observed *EGFR* mutations in NSCLC.^19,21,22,25^ *EGFR* exon 20 insertion mutations are largely mutually exclusive with other known oncogenic driver events that are characteristic of NSCLC, such as *KRAS* mutations,^26^ and follow similar trends as classical activating *EGFR* mutations; *EGFR* exon 20 insertion mutations are enriched in women, non-smokers, Asian populations, and those with adenocarcinoma histology.^25^

Until recently, *EGFR* exon 20 insertion mutations have been exclusively reported in NSCLC. Interestingly, however, recent analysis of a rare form of head and neck cancer known as sinonasal squamous cell carcinoma (SNSCC) demonstrated a remarkably high frequency of *EGFR* mutations (77% of SNSCC tumors), the majority of which were exon 20 insertions (88% of all *EGFR* mutations).^27^ This result is striking given that *EGFR* mutations in head and neck cancer are rare overall, and moreover, the frequency of *EGFR* mutations is inverse to that of NSCLC; in SNSCC tumors, exon 20 insertion mutations predominate, while exon 19 deletions are detected at low frequency.^27,28^ Thus, *EGFR* exon 20 insertions are not entirely restricted to lung cancer as previously believed but can also act as oncogenic drivers in SNSCC, which represents approximately 3% of all head and neck cancers.^29,30^

Notably, structurally analogous exon 20 insertion mutations that can promote ligand-independent activation that are also found in *HER2*, another member of the EGFR family of receptor tyrosine kinases (RTK).^23,31^ Although *HER2* mutations are present at a much lower frequency (~2% of NSCLC patients) compared with *EGFR* mutations, exon 20 insertions are, by far, the most dominant type of *HER2* aberration in NSCLC, representing greater than 90% of all observed *HER2* mutations.^32,33^

## Impact of exon 20 insertions on EGFR structure, activity, and sensitivity to EGFR inhibitors

EGFR was the first RTK to be identified and belongs to a subset of RTKs known as the EGFR family.^34,35^ For wild-type EGFR, ligand binding is required for activation and induces a conformational change that facilitates receptor homo-dimerization or hetero-dimerization with other EGFR family members. Subsequent autophosphorylation of the tyrosine residues in the intracellular tail of EGFR initiates the formation of large protein complexes that can propagate downstream signaling.^36–38^ Many of the signaling pathways activated downstream of EGFR, including the Ras/Raf/Mitogen-activated protein kinase (Ras/MAPK) pathway, phosphatidylinositol 3-kinase/AKT (PI3K/AKT) pathway, and signal transducers and activators of transcription (STAT) pathway orchestrate key cellular processes including cell survival, proliferation, and migration. As such, it follows that dysregulation of EGFR has the potential to contribute to almost all of the classical oncogenic phenotypes that have been described as the ‘hallmarks of cancer’.^39^

The conformational changes induced by the L858R mutation and exon 19 deletions have been predicted to destabilize the inactive form of EGFR, causing an overall equilibrium shift towards an active over an inactive state.^40–42^ This shift allows ligand-independent dimerization and activation of the receptor, resulting in constitutive activation of downstream signaling pathways. In lung cancers with classical *EGFR* mutations, blockade of EGFR signaling with inhibitors can trigger rapid apoptosis in a manner consistent with the ‘oncogene addiction’ model, in which cells are dependent on persistent EGFR signaling for survival.^43^ Importantly, kinetic analyses have revealed that both L858R and exon 19 deletion EGFR kinases display a greatly reduced affinity for ATP relative to the wild-type receptor.^44,45^ Diminished ATP binding has the opposite effect on drug binding; the relative affinity of mutant receptors for reversible ATP-competitive EGFR inhibitors, such as gefitinib or erlotinib, is potently enhanced by alleviating the competitive pressure. This differential sensitivity between wild-type and mutant receptors, combined with the increased dependency of tumor cells on EGFR signaling, provides the wide therapeutic window that makes EGFR inhibitor therapy much more effective in patients with classical activating *EGFR* mutations.

A second mutational event in *EGFR*, the T790M substitution in exon 20, is thought to account for more than half of all cases of acquired resistance to first-generation EGFR inhibitors in NSCLC.^46^ It was initially predicted that the mechanism of resistance underlying the T790M mutation was steric hindrance imposed by the presence of a bulky methionine residue that would prevent the binding of first-generation EGFR inhibitors to EGFR. However, while the T790M mutation was found to impact the affinity of the mutant EGFR receptor to gefitinib, inhibitor binding was not completely abolished.^47^ Crucially, further analysis revealed another major factor that contributed to drug resistance: the T790M mutation restored the ATP-binding affinity of the L858R mutant *EGFR* to almost wild-type receptor levels.^40,47^ By increasing the ATP affinity, the T790M mutation diminishes the efficacy of reversible ATP-competitive inhibitors gefitinib and erlotinib, and removes the selectivity that these drugs have for mutant over wild-type EGFR.

**Fig. 3:**
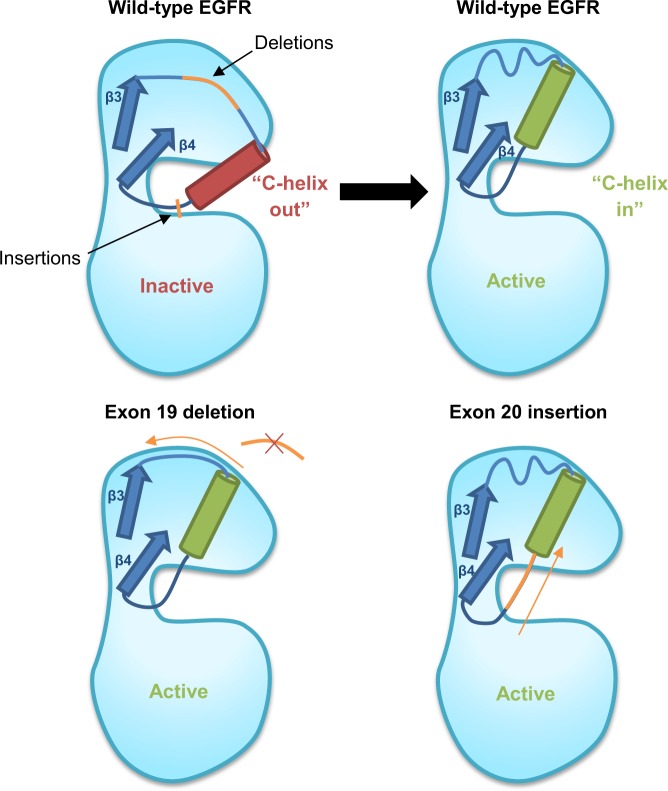
Impact of deletions and insertions on EGFR activation. Upon ligand-binding, the regulatory C-helix pivots from an outward, inactive conformation to an inward, active conformation to form key interactions with the p-loop of the active site located in the cleft between the N-lobe and C-lobe. Oncogenic mutations such as exon 19 deletions can “pull” the C-helix from the N-terminal side whilst exon 20 insertions “push” from the C-terminal side to stabilize the active state of EGFR even in the absence of ligand

Similarly, the unique mechanism of EGFR activation induced by exon 20 insertions underlies the relative resistance of these mutations to clinically approved EGFR inhibitors. Exon 20 insertions are positioned towards the C-terminal end of a specific structural component of EGFR known as the C-helix. The C-helix is a key regulatory element that dictates the activation status of EGFR by rotating from an outward to an inward position, permitting specific interactions with the active site that stabilize dimerization-competent EGFR.^40^ Exon 19 deletions in *EGFR* affect this region via removal of residues from the loop that leads up to the C-helix on the N-terminal side (Fig. 3). The shortening of this loop as a result of exon 19 deletions has been hypothesized to “pull” and restrict the rotation of the C-helix, preventing it from adopting the outward, inactive conformation and favoring the inward, active conformation. It is by this mechanism that exon 19 deletion mutations have been predicted to exert the equilibrium shift towards active over inactive EGFR states, promoting constitutive receptor activation. Conversely, exon 20 insertions at the opposite C-terminal end of the C-helix, or much more commonly in the loop that immediately follows it, have been predicted to “push” the C-helix into an active conformation from the other direction.^40^

In 2013, Yasuda et al. solved the first crystal structure of a representative exon 20 insertion *EGFR* mutation, D770_N771insNPG, which provided valuable insights into the mechanism of EGFR activation and drug resistance associated with exon 20 insertion mutations.^22^ The crystal structure of D770_N771insNPG revealed an active conformation with the C-helix adopting an inward position. The position of the insertion formed a wedge at the “pivot point” of the C-helix, creating a rigid, inflexible structure that likely prevents the reorientation of the C-helix to the outward, inactive state, confirming previous predictions. In this manner, exon 20 insertions essentially “lock” EGFR molecules in an active conformation in the absence of ligand binding. Further analysis of the crystal structure together with in vitro kinetic studies performed on purified kinase domains provided an explanation for the insensitivity of exon 20 insertion mutations to EGFR inhibitors. Yasuda et al. observed that the D770_N771insNPG mutation is capable of activating EGFR without markedly diminishing ATP affinity or enhancing affinity for the first-generation inhibitor gefitinib.^22^ Thus, the two key features that confer the exquisite sensitivity of L858R and exon 19 deletion EGFR mutations to first-generation EGFR inhibitors are absent in the presence of exon 20 insertions, and the therapeutic window to target mutant over wild-type EGFR is lost. A later study used computational modeling of the solved D770_N771insNPG crystal structure to attribute the lack of affinity for first-generation EGFR inhibitors to a prominent shift of the C-helix and phosphate-binding loop (p-loop) of EGFR into the drug-binding pocket, resulting in significant steric hindrance.^31^

It is important to note that *EGFR* exon 20 insertion mutations are heterogeneous and that the location of the insertion, in particular, may influence the kinetics of drug and ATP binding, ultimately determining resistance or sensitivity to EGFR inhibitors. Indeed, Yasuda et al. also investigated another exon 20 insertion that occurred within the C-helix of EGFR, A763_Y764insFQEA, and unexpectedly found that this mutation had a high affinity for gefitinib in vitro and was highly sensitive to erlotinib in an engineered cell line model.^22^ Three patients with the A763_Y764insFQEA insertion showed tumor regression or stable disease following erlotinib treatment, leading the authors to conclude that unlike other mutations of its class, this particular exon 20 insertion is sensitive to first-generation EGFR inhibitors. Although its crystal structure was not solved, 3D modeling of the A763_Y764insFQEA insertion suggests that exon 20 insertions that occur before residue 764, within the C-helix itself, might have an activation mechanism and structure that more closely resemble those of the L858R or exon 19 deletion mutant *EGFR* and therefore would be predicted to be sensitive to first-generation EGFR inhibitors. In agreement, multiple reports have shown partial responses of patients harboring A763_Y764insFQEA insertions to the first-generation inhibitor erlotinib, indicating that these mutations may behave similarly to classical NSCLC *EGFR* mutations.^19,48^ An outstanding question is whether the downstream signaling pathways activated by distinct *EGFR* exon 20 insertion mutations are the same and to what degree these pathways overlap with those activated by the more common L858R or exon 19 deletion *EGFR* mutations. Previous work has shown that distinct EGFR mutations drive unique downstream signaling profiles.^49–51^ Future characterization of the downstream pathway activation that is unique to *EGFR* exon 20 insertions may yield mutation-specific therapies that selectively target these pathways.

## Treatment options for *EGFR* exon 20 insertion NSCLC

### First-generation EGFR inhibitors

Commonly referred to as the ‘first-generation’ EGFR inhibitors, gefitinib and erlotinib are reversible, ATP-competitive inhibitors that selectively target exon 19 deletion and L858R mutant EGFR over wild-type and have been the gold standard of *EGFR*-mutant positive NSCLC treatment, achieving up to a 72% RR and nearly 10 months PFS.^4,16,17^ By contrast, with the exception of the A763_Y764insFQEA mutation,^19,48^ retrospective analysis of clinical data has revealed that first-generation EGFR inhibitors are ineffective in the vast majority of *EGFR* exon 20 insertion mutant NSCLC patients, with a RR between 8 and 27% and less than 3 months median PFS reported (Table 1).^52,53^

### Second-generation EGFR inhibitors

Three second-generation EGFR inhibitors have been developed: neratinib, dacomitinib, and afatinib. Unlike the first-generation drugs gefitinib or erlotinib, all of these compounds form irreversible, covalent interactions with EGFR at a specific cysteine residue (C797) and have additional activity against at least one other EGFR family member.^54^ A phase II clinical trial (NCT00266877) of neratinib reported a low RR of only 3% in classical *EGFR* mutant positive lung adenocarcinoma tumors, and there are currently no ongoing clinical trials to assess neratinib in lung cancer.^55^ An ongoing phase III clinical trial, ARCHER 1050 (NCT01774721), has recently demonstrated significantly longer median PFS after dacomitinib treatment (14.7 months) versus gefitinib treatment (9.2 months) in NSCLC patients harboring classical EGFR mutations, leading to FDA approval for dacomitinib to treat metastatic EGFR mutant-positive NSCLC in September 2018.^56^ Afatinib is also approved as a first-line therapy for the treatment of NSCLC harboring classical *EGFR* mutations based on two pivotal studies that demonstrated a significant PFS benefit of greater than 10 months in *EGFR*-mutant patients.^57,58^

Currently, there is limited clinical evidence that supports the use of second-generation EGFR inhibitors in exon 20 insertion mutant NSCLC. A recent post-hoc analysis of data from 100 lung cancer patients harboring rare *EGFR* mutations pooled from LUX-Lung 2, LUX-Lung 3, and LUX-Lung 6 trials revealed significant clinical activity of afatinib (71.1% RR, 10.7 months PFS) in rare *EGFR* point mutations (G719X, S786I, L861Q).^59^ This finding led the FDA to broaden the indication for afatinib to include NSCLC patients who harbor these specific mutations in January 2018. However, in the same analysis, a group of 23 patients harboring *EGFR* exon 20 insertions treated with afatinib were found to have a minimal response (8.7% RR, 2.7 months PFS).

Interestingly, a comprehensive pre-clinical study of exon 20 insertions in *EGFR* and *HER2* in engineered Ba/F3 and NIH-3T3 cell line models has identified a variable degree of responses to the second-generation inhibitors dacomitinib, neratinib, and afatinib across distinct exon 20 insertions.^60^ In particular, the authors noted that insertions that introduced a glycine at position 770 were uniquely sensitive to the EGFR/HER2 inhibitor dacomitinib, suggesting a potential therapeutic avenue for patients with mutations that share this feature. In a 2011 phase I clinical trial, out of 6 patients with different *EGFR* exon 20 insertion mutations, the only patient who demonstrated a partial response to dacomitinib harbored a D770delinsGY mutation, supporting the pre-clinical data.^61^ However, these mutations are relatively rare—the most common exon 20 insertions lack glycine at position 770; therefore, the majority of patients within this NSCLC molecular subgroup are unlikely to benefit from dacomitinib treatment. Consistent with the clinical data, the pre-clinical study found that only the cell line models expressing a D770delinsGY mutation possessed an in vitro IC_50_ that was sufficiently low enough (17.5 nM) to predict a response to dacomitinib in patients based on current dosing regimens (a 45 mg once per day dose, which is an approximately 120–160 nmol/L trough concentration).^62^

### Third-generation EGFR inhibitors

In response to the identification of the T790M secondary mutation as a major source of resistance to first-generation EGFR inhibitors in NSCLC, third-generation EGFR inhibitors that covalently bind to the C797 cysteine residue of EGFR and maintain selectivity for the double mutant L858R/T790M or exon 19 del/T790M EGFR have been developed.^46,63,64^ Two major T790M-selective irreversible EGFR inhibitors, rociletinib, and osimertinib, have been assessed in the context of *EGFR* T790M-mutant positive NSCLC, and based on its impressive response rate of greater than 60%, the FDA granted approval for osimertinib as a second-line treatment for *EGFR* T790M mutant-positive patients following progression on erlotinib or gefitinib in 2017.^8,9,65^ Recently, osimertinib has also demonstrated a significant benefit versus first-generation EGFR inhibitors in previously untreated NSCLC patients harboring classical *EGFR* mutations. In 556 *EGFR*-mutant patients, osimertinib-treated patients reached an unprecedented median PFS of 17.2 months versus 8.5 months with first-generation EGFR inhibitor treatment, leading to its recent approval as a first-line therapy.^66^

Pre-clinical in vitro evidence in engineered cell line models has suggested that osimertinib may have some activity against *EGFR* exon 20 insertions, albeit with a weaker potency than afatinib.^31,67^ However, the evidence to support osimertinib as a candidate inhibitor for *EGFR* exon 20 insertions in vivo remains unclear. A study of lung cancer patient-derived xenograft (PDX) models harboring *EGFR* exon 20 insertion mutations showed poor responses to the third-generation EGFR inhibitors osimertinib and rociletinib.^68^ However, Floc’h et al. have recently demonstrated significant antitumor activity of both osimertinib and its circulating metabolite AZ5104 using xenograft models of the H2073 *EGFR* wild-type lung cancer cell line, which was genetically engineered to introduce either the D770_N771InsSVD or V769_D770InsASV *EGFR* insertion mutations.^69^ A phase II clinical trial to assess osimertinib as a treatment for *EGFR* exon 20 insertion mutant NSCLC (NCT03414814) is ongoing.

### Poziotinib

Poziotinib (formerly HM781–36B) is a covalent, irreversible inhibitor of EGFR and HER2^70^ that is currently the most advanced clinical candidate of compounds with the capacity to target *EGFR* exon 20 insertions. Initially, a phase II study of poziotinib in NSCLC patients with classical *EGFR* mutations who had acquired resistance to EGFR inhibitors via the T790M mutation or other mechanisms found that poziotinib had minimal clinical activity.^71^ Poziotinib was subsequently shown to have significant activity against EGFR exon 20 insertions in in vitro models, a result which was mirrored by encouraging clinical data from an ongoing phase II trial (NCT03066206).^31^ Robichaux et al. used 3D modeling to demonstrate how the rigid C-helix conformation induced by exon 20 insertions results in a relatively small drug binding pocket. This restricted conformation has been predicted to prevent the binding of drugs such as osimertinib, which has a large terminal group and a rigid pyrimidine core, by steric hindrance. By contrast, poziotinib is centered on a less rigid quinazoline core, akin to second-generation EGFR inhibitors. In addition, poziotinib has small terminal and substituent linking groups, making it more compact and flexible compared to current second-generation and third-generation inhibitors. Based on these features, 3D modeling predicts that poziotinib is able to tightly bind the restricted exon 20 insertion binding pocket of EGFR and may also be effective against structurally analogous exon 20 insertions in HER2.

Following these structural modeling studies, the authors demonstrated potent inhibition of exon 20 insertion EGFR and HER2 mutants in a series of engineered Ba/F3 cell line models. In these cell lines, poziotinib was shown to be uniquely selective for exon 20 insertions over T790M mutants, providing a possible explanation for the initial disappointing results in patients with acquired resistance to EGFR inhibitors in the phase II trial.^71^ However, it is important to note that poziotinib also demonstrated activity against wild-type EGFR in vitro, highlighting a possible narrow therapeutic window that may give rise to dose limitations in *EGFR* exon 20 insertion patients. Using genetically engineered mouse models harboring *EGFR* D770insNPG or *HER2* A775insYVMA lung tumors, a durable response to poziotinib was shown over 12 weeks of treatment in vivo. Additionally, poziotinib was compared to afatinib in a mouse xenograft model of a patient-derived cell line, YUL-0019, which harbors EGFR N771delinsFH. Although YUL-0019 tumors did not grow in the presence of afatinib, tumor volume remained stable over the course of 10 days. By contrast, poziotinib treatment resulted in a 50% reduction of tumor volume within the same time frame.

The authors also reported preliminary data from an ongoing phase II clinical trial for poziotinib in *EGFR* exon 20 insertion-positive lung cancer patients (NCT03066206). Although the patient cohort was small, a striking 64% confirmed response rate was achieved in 11 patients harboring *EGFR* exon 20 insertion mutations. This promising result is in stark contrast to the 8.7% response rate that has previously been observed for afatinib in exon 20 insertion patients.^59^ It is important to note, however, that the clinical data from this trial are not mature and the median PFS and OS have not yet been reported. Therefore, a question remains: how durable will the response to poziotinib be in patients with exon 20 EGFR insertions? Anticipating the emergence of acquired resistance, Robichaux et al. indirectly showed a possible mechanism that parallels osimertinib resistance by demonstrating that Ba/F3 cells expressing EGFR L858R/T790M/C797S or EGFR exon 19 deletion/T790M/C797S triple mutants are resistant to poziotinib treatment in vitro. The C797S *EGFR* mutation has been found to be a major clinical mechanism of acquired resistance to osimertinib treatment in patients with classical *EGFR* mutants who are concurrently T790M positive.^72^ However, whether the C797S mutation arises in patients with exon 20 insertion mutations has yet to be determined. Similarly, whether poziotinib is clinically effective across a broad spectrum of different EGFR exon 20 insertions with sufficient selectivity over wild-type EGFR to achieve target inhibition with minimal toxicity remains unknown. Based on preliminary results from a phase II clinical trial including data from a small cohort of 30 patients, poziotinib was recently declined Breakthrough Designation Therapy status by the FDA,^73^ although the trial remains ongoing to assess poziotinib in a larger cohort of NSCLC patients harboring *EGFR* or *HER2* exon 20 insertions (NCT03318939).

### Luminespib

Luminespib is an inhibitor of heat shock protein 90 (Hsp90), a molecular chaperone required to maintain stability and assist protein folding for a large number of cellular client proteins, including receptors and signaling components involved in driving oncogenesis.^74^ Importantly, Hsp90 inhibitors have shown anti-tumor properties and selectivity for cancer cells over normal cells,^75^ and although no Hsp90 inhibitors have been approved to date, their potential is being actively explored across many cancer types for which treatment options are limited.^76^ Recently, pre-clinical data have shown that the EGFR exon 20 insertion mutant kinase associates with the Hsp90 chaperone system and can be degraded through the use of Hsp90 inhibitors.^77^ A phase II clinical trial of luminespib in *EGFR* exon 20 insertion NSCLC (NCT01854034) reported that 5 out of 29 (17%) patients achieved partial or complete responses, indicating an improvement over the current RRs to first-generation or second-generation EGFR inhibitors.^78^ However, both the median PFS (2.9 months) and overall survival (13 months) were short, while the ocular toxicity of this class of drugs remains a concern. Therefore, it is unclear at this stage whether luminespib will have clinical utility in *EGFR* exon 20 insertion NSCLC patients.

### Cetuximab and EGFR inhibitor combinations

Based on in silico structural modeling, two *EGFR* exon 20 insertions, D770_P772del_insKG and D770>GY, were predicted to increase the electrostatic energy between EGFR monomers and therefore favor the formation of EGFR active dimers.^79^ This finding led the authors to hypothesize that patients harboring these insertions may be sensitive to a combination of EGFR kinase inhibitors and cetuximab, a monoclonal antibody that binds to the extracellular domain of EGFR and sterically hinders dimer formation.^80^ Case studies from two patients support the use of cetuximab in combination with EGFR inhibitors or chemotherapy, including one patient (D770>GY) treated with a combination of cetuximab and erlotinib in a phase I clinical trial with a reported PFS of 3.5 years.^79,81^ More recently, a clinical study found that three out of four *EGFR* exon 20 insertion-positive NSCLC patients had partial responses to a combination of afatinib and cetuximab, with a median PFS of 5.4 months.^82^ These results suggest that the cetuximab and EGFR inhibitor combination may have some efficacy in patients with exon 20 insertions; however, with limited clinical data, further work is necessary to determine the impact of the insertion size and location on the response to cetuximab combinations.

### Tarloxotinib

Tarloxotinib is a hypoxia-activated prodrug (HAP) that releases an irreversible EGFR/HER2 inhibitor only under low oxygen conditions.^83,84^ Hypoxia is a common feature of solid tumors and, in particular, has been linked to EGFR inhibitor resistance in pre-clinical models.^85–87^ A phase II clinical trial of tarloxotinib in EGFR mutant, T790M-negative NSCLC patients with progression on first-line EGFR inhibitors reported disappointing results; while 7 of 21 patients achieved stable disease, no confirmed partial responses were reported, leading to early termination of the trial.^84,88^ However, a recent in vivo study using murine xenografts of two NSCLC cell lines harboring endogenous *EGFR* exon 20 insertions, CUTO14 (A767_V769dupASV) and CUTO17 (N771_H773dupNPH), has revealed significant tumor regression with tarloxotinib treatment compared with the lack of a tumor response to afatinib.^89^ These preliminary data suggest that more focused clinical trials are warranted to determine the efficacy of tarloxotinib within the *EGFR* exon 20 insertion NSCLC patient subgroup.

## *EGFR* exon 20 insertion selective inhibitors

The development of compounds with selectivity for EGFR exon 20 insertion mutants over wild-type EGFR is crucial to limit the toxicity that arises from wild-type EGFR inhibition in patients.^90^ Several novel inhibitor compounds have recently been developed and have been shown to directly target the EGFR exon 20 insertion mutant kinase. Although these developments remain at an early stage, published data suggest that these compounds may have significant clinical activity in NSCLC patients harboring exon 20 insertion mutations in *EGFR* and *HER2*. In this section, we will discuss the latest pre-clinical evidence that supports the evaluation of these *EGFR* exon 20 insertion selective inhibitors in lung cancer patients.

### TAK-788

TAK-788 (formerly AP32788) is a covalent, irreversible inhibitor that is designed to selectively target the exon 20 insertion mutant forms of both EGFR and HER2 kinases over wild-type EGFR.^91^ Gonzalvez et al. reported selective activity of TAK-788 against 14 different *EGFR* exon 20 insertion mutant variants expressed in the Ba/F3 cell line model. In the same study, tumor regression was observed in a patient-derived xenograft NSCLC model harboring an *EGFR* exon 20 insertion mutation following once daily dosing of TAK-788. A phase I/II clinical trial (NCT02716116) is now underway to evaluate the use of TAK-788 in NSCLC patients with exon 20 insertion mutations in *EGFR* or *HER2*. A preliminary first report of the dose-escalation cohort demonstrated tolerability of TAK-788 in patients, with an adverse effect profile similar to that of other EGFR inhibitors used in NSCLC.^92^ Once a maximum tolerated dose is established, the phase II expansion phase aims to determine the overall response rate to TAK-788 in a larger cohort of NSCLC patients harboring *EGFR* or *HER2* exon 20 insertions either with or without brain metastases.

### TAS6417

TAS6417 (formerly TPC-064) is a newly developed irreversible EGFR inhibitor that was designed to fit into the ATP binding site of the exon 20 insertion EGFR kinase.^93^ Mass spectrometry analysis demonstrated that TAS6417 forms a covalent interaction with an insertion mutant EGFR kinase at the cysteine residue C797. Further pre-clinical characterization of TAS6417 by Hasako et al. demonstrated selectivity for the D770_N771insNPG mutant EGFR kinase over wild-type EGFR using cell-free in vitro kinase assays. To ensure cellular activity, TAS6417 treatment was shown to inhibit EGFR phosphorylation and cell viability in NIH3T3 cell line models engineered to express a panel of 6 distinct *EGFR* exon 20 insertion mutations. In addition, as an elegant solution to the paucity of NSCLC cell lines harboring endogenous *EGFR* exon 20 insertion mutations, the authors employed transcription activator-like effector nuclease (TALEN) mutagenesis to generate a suitable model derived from the H1975 *EGFR* L858R/T790M mutant NSCLC cell line. TALEN mutagenesis was used to introduce an *EGFR* D770_N771insSVD insertion into the H1975 cell line and subsequently knock out endogenous L858R and T790M mutations. TAS6417 treatment was then shown to inhibit cell growth of the H1975 *EGFR* D770_N771insSVD mutant cell line in vitro and in vivo. It was also confirmed that TAS6417 treatment resulted in the inhibition of EGFR phosphorylation in xenografts, coupled with evidence of inhibition of two critical pathways that mediate oncogenic signaling downstream of EGFR, the PI3K/Akt (as measured by pAKT) and Ras/MAPK (as measured by pERK) pathways. Moreover, the level of EGFR signaling inhibition correlated with the plasma concentrations of TAS6417 over a 24 h time course of treatment. Clinical assessment of TAS6417 in NSCLC patients has not yet begun; therefore, it remains to be determined whether the mutant selectivity of the inhibitor is sufficient to achieve a good therapeutic window with low toxicity in *EGFR* exon 20 insertion-positive patients.

### Compound 1A

Jang et al. have recently employed a structure-guided rational drug design strategy to develop a currently unnamed lead compound, referred to as ‘compound 1A’, which has activity against EGFR and HER2 exon 20 insertion mutant kinases.^94^ Taking inspiration from EGFR inhibitors that form covalent interactions with EGFR, such as osimertinib and rociletinib,^64,95^ compounds were designed using a similar pyrimidine core structure that had the ability to bind the cysteine residue C797 of EGFR. The authors hypothesized that small molecule compounds that can form additional molecular interactions with the active conformation of EGFR would have enhanced potency against the EGFR exon 20 insertion mutant kinase. By examining the crystal structure of wild-type EGFR complexed with osimertinib, Jang et al. observed a deep hydrophobic pocket of EGFR positioned at the back of the ATP-binding site that was not occupied by osimertinib. Based on the original pyrimidine core, a novel series of chemical compound analogues were generated by the addition of substituents that were intended to interact with EGFR in the deep hydrophobic pocket. In particular, the compound identified as ‘1A’ was found to strongly inhibit EGFR phosphorylation in Ba/F3 cell lines expressing *EGFR* exon 20 insertion mutations and demonstrated less potency against wild-type EGFR compared with the second-generation EGFR inhibitors afatinib and dacomitinib and the EGFR exon 20 targeting compound poziotinib. Jang et al. also showed potent antiproliferative activity against a patient-derived NSCLC cell line, DFCI127, which harbors an EGFR P772_H773insPNP mutation. The poor pharmacokinetic properties of compound 1A, including its low oral bioavailability, short half-life, and high clearance rate, remain to be addressed before assessing the efficacy of this inhibitor in in vivo models.

## Conclusions

To date, clinically approved targeted EGFR inhibitors have failed to effectively treat NSCLC driven by *EGFR* exon 20 insertion mutations.^19^ Crystal structures and in vitro analyses of exon 20 insertion EGFR mutant kinases have revealed that, unlike L858R or exon 19 deletion mutant EGFR, the majority of exon 20 insertions do not possess diminished ATP binding or enhanced affinity for first-generation EGFR inhibitors over wild-type EGFR, thereby reducing the therapeutic window required for their clinical benefit.^22^ Compounds with the ability to overcome these issues and target *EGFR* exon 20 insertion mutations, therefore, present an exciting opportunity for clinical use in this patient population. For example, the most advanced clinical compound to date, poziotinib, has achieved a 64% objective response rate, a significant improvement upon the previous 8.7% objective response rate for afatinib^59^ and 17% objective response rate for the Hsp90 inhibitor luminespib.^96^ Several outstanding challenges lie ahead–for instance, given the significant molecular heterogeneity of the size and location of distinct *EGFR* exon 20 insertions, it will be important to determine whether all patients within this molecular subtype of NSCLC will universally respond to these inhibitors. Moreover, the long-term clinical durability of *EGFR* exon 20 insertion-selective drugs remains unclear, and identifying eventual resistance mechanisms and strategies to overcome them will be crucial in achieving durable patient responses. Considerable efforts are underway to explore the concept of synthetic lethality to identify additional dependencies in classical mutant EGFR-driven models that can be targeted to overcome drug resistance.^97^ An unresolved question is whether *EGFR* exon 20 insertion-driven NSCLC will share similar downstream signaling profiles and vulnerabilities that can be exploited therapeutically. Nevertheless, based on encouraging preliminary data with *EGFR* exon 20 selective compounds, it is anticipated that one or more of these inhibitors will achieve good tolerability and clinical efficacy in patients that will lead to regulatory approval. Moving forward, there is the exciting prospect that these selective kinase inhibitors will provide novel and effective treatment options for patients with *EGFR* exon 20 insertion mutant-positive NSCLC in the near future.

## References

[1] Ferlay, J. et al. GLOBOCAN 2012v1.0, Cancer incidence and mortality worldwide: IARC CancerBase. No. 11. *Lyon* (International Agency for Research on Cancer, France, 2013).

[2] Molina JR, Yang P, Cassivi SD, Schild SE, Adjei AA (2008). Non-small cell lung cancer: epidemiology, risk factors, treatment, and survivorship. Mayo Clin. Proc..

[3] Zhou C (2011). Erlotinib versus chemotherapy as first-line treatment for patients with advanced EGFR mutation-positive non-small-cell lung cancer (OPTIMAL, CTONG-0802): a multicentre, open-label, randomised, phase 3 study. Lancet Oncol..

[4] Rosell R (2012). Erlotinib versus standard chemotherapy as first-line treatment for European patients with advanced EGFR mutation-positive non-small-cell lung cancer (EURTAC): a multicentre, open-label, randomised phase 3 trial. Lancet Oncol..

[5] Solomon BJ (2014). First-line crizotinib versus chemotherapy in *ALK-*positive lung cancer. N. Engl. J. Med..

[6] Planchard D (2017). Dabrafenib plus trametinib in patients with previously untreated BRAFV600E-mutant metastatic non-small-cell lung cancer: an open-label, phase 2 trial. Lancet Oncol..

[7] Shaw AT (2014). Crizotinib in *ROS1* -rearranged non–small-cell lung cancer. N. Engl. J. Med..

[8] Yang JCH (2017). Osimertinib in pretreated T790M-positive advanced non-small-cell lung cancer: AURA study phase II extension component. J. Clin. Oncol..

[9] Goss G (2016). Osimertinib for pretreated EGFR Thr790Met-positive advanced non-small-cell lung cancer (AURA2): a multicentre, open-label, single-arm, phase 2 study. Lancet Oncol..

[10] Shepherd FA (2005). Erlotinib in previously treated non-small-cell lung cancer. N. Engl. J. Med..

[11] Fukuoka M (2003). Multi-institutional randomized phase ii trial of gefitinib for previously treated patients with advanced non-small-cell lung cancer. J. Clin. Oncol..

[12] Kris MG (2003). Efficacy of gefitinib, an inhibitor of the epidermal growth factor receptor tyrosine kinase, in symptomatic patients with non-small cell lung cancer: a randomized trial. JAMA.

[13] Lynch TJ (2004). Activating mutations in the epidermal growth factor receptor underlying responsiveness of non-small-cell lung cancer to gefitinib. N. Engl. J. Med..

[14] Paez JG (2004). EGFR mutations in lung cancer: correlation with clinical response to gefitinib therapy. Science.

[15] Pao W (2004). EGF receptor gene mutations are common in lung cancers from ‘never smokers’ and are associated with sensitivity of tumors to gefitinib and erlotinib. Proc. Natl Acad. Sci. USA.

[16] Hirsch FR (2006). Molecular predictors of outcome with gefitinib in a phase III placebo-controlled study in advanced non-small-cell lung cancer. J. Clin. Oncol..

[17] Mok TS (2009). Gefitinib or carboplatin–paclitaxel in pulmonary adenocarcinoma. N. Engl. J. Med..

[18] Gazdar AF (2009). Activating and resistance mutations of EGFR in non-small-cell lung cancer: role in clinical response to EGFR tyrosine kinase inhibitors. Oncogene.

[19] Arcila ME (2013). EGFR exon 20 insertion mutations in lung adenocarcinomas: prevalence, molecular heterogeneity, and clinicopathologic characteristics. Mol. Cancer Ther..

[20] Kobayashi Y, Mitsudomi T (2016). Not all epidermal growth factor receptor mutations in lung cancer are created equal: Perspectives for individualized treatment strategy. Cancer Sci..

[21] Yasuda H, Kobayashi S, Costa DB (2012). EGFR exon 20 insertion mutations in non-small-cell lung cancer: Preclinical data and clinical implications. Lancet Oncol..

[22] Yasuda H (2013). Structural, biochemical, and clinical characterization of epidermal growth factor receptor (EGFR) exon 20 insertion mutations in lung cancer. Sci. Transl. Med..

[23] Kosaka T (2004). Mutations of the epidermal growth factor receptor gene in lung cancer: biological and clinical implications. Cancer Res..

[24] Shigematsu H (2005). Clinical and biological features associated with epidermal growth factor receptor gene mutations in lung cancers. J. Natl Cancer Inst..

[25] Oxnard GR (2013). Natural history and molecular characteristics of lung cancers harboring EGFR exon 20 insertions. J. Thorac. Oncol..

[26] Riess JW (2018). Diverse EGFR Exon 20 insertions and co-occurring molecular alterations identified by comprehensive genomic profiling of NSCLC. J. Thorac. Oncol..

[27] Udager AM (2015). High-frequency targetable EGFR mutations in sinonasal squamous cell carcinomas arising from inverted sinonasal papilloma. Cancer Res..

[28] Stransky N (2011). The mutational landscape of head and neck squamous cell carcinoma. Science.

[29] Lewis JS, Jr. (2016). Sinonasal squamous cell carcinoma: a review with emphasis on emerging histologic subtypes and the role of human papillomavirus. Head Neck Pathol..

[30] Ansa B (2013). Paranasal sinus squamous cell carcinoma incidence and survival based on surveillance, epidemiology, and end results data, 1973 to 2009. Cancer.

[31] Robichaux JP (2018). Mechanisms and clinical activity of an EGFR and HER2 exon 20-selective kinase inhibitor in non-small cell lung cancer. Nat. Med..

[32] Arcila ME (2012). Prevalence, clinicopathologic associations, and molecular spectrum of ERBB2 (HER2) tyrosine kinase mutations in lung adenocarcinomas. Clin. Cancer Res..

[33] Mazieres J (2013). Lung cancer that harbors an HER2 mutation: epidemiologic characteristics and therapeutic perspectives. J. Clin. Oncol..

[34] Carpenter G, King LJ, Cohen S (1978). Epidermal growth factor stimulates phosphorylation in membrane preparations in vitro. Nature.

[35] Lemmon MA, Schlessinger J (2010). Cell signaling by receptor tyrosine kinases. Cell.

[36] Lowenstein EJ (1992). The SH2 and SH3 domain-containing protein GRB2 links receptor tyrosine kinases to ras signaling. Cell.

[37] Yarden Y, Sliwkowski MX (2001). Untangling the ErbB signalling network. Nat. Rev. Mol. Cell Biol..

[38] Scaltriti M, Baselga J (2006). The epidermal growth factor receptor pathway: a model for targeted therapy. Clin. Cancer Res..

[39] Hanahan D, Weinberg RA (2011). Hallmarks of cancer: the next generation. Cell.

[40] Eck MJ, Yun CH (2010). Structural and mechanistic underpinnings of the differential drug sensitivity of EGFR mutations in non-small cell lung cancer. Biochim. Et. Biophys. Acta.

[41] Yun CH (2007). Structures of lung cancer-derived EGFR mutants and inhibitor complexes: mechanism of activation and insights into differential inhibitor sensitivity. Cancer Cell..

[42] Landau M, Ben-Tal N (2008). Dynamic equilibrium between multiple active and inactive conformations explains regulation and oncogenic mutations in ErbB receptors. Biochim. Biophys. Acta.

[43] Weinstein IB, Joe A (2008). Oncogene addiction. Cancer Res..

[44] Carey KD (2006). Kinetic analysis of epidermal growth factor receptor somatic mutant proteins shows increased sensitivity to the epidermal growth factor receptor tyrosine kinase inhibitor, erlotinib. Cancer Res..

[45] Mulloy R (2007). Epidermal growth factor receptor mutants from human lung cancers exhibit enhanced catalytic activity and increased sensitivity to gefitinib. Cancer Res..

[46] Kosaka T (2006). Analysis of epidermal growth factor receptor gene mutation in patients with non-small cell lung cancer and acquired resistance to gefitinib. Clin. Cancer Res..

[47] Yun CH (2008). The T790M mutation in EGFR kinase causes drug resistance by increasing the affinity for ATP. Proc. Natl Acad. Sci..

[48] Voon PJ, Tsui DWY, Rosenfeld N, Chin TM (2013). Letter to Editor: EGFR Exon 20 Insertion A763-Y764insFQEA and response to Erlotinib. Mol. Cancer Ther..

[49] Huang PH, Xu AM, White FM (2009). Oncogenic EGFR signaling networks in glioma. Rev. Lit. Arts Am..

[50] Pines G, Huang PH, Zwang Y, White FM, Yarden Y (2010). EGFRvIV: a previously uncharacterized oncogenic mutant reveals a kinase autoinhibitory mechanism. Oncogene.

[51] Guha U (2008). Comparisons of tyrosine phosphorylated proteins in cells expressing lung cancer-specific alleles of EGFR and KRAS. Proc. Natl Acad. Sci..

[52] Naidoo J (2015). Epidermal growth factor receptor exon 20 insertions in advanced lung adenocarcinomas: clinical outcomes and response to erlotinib. Cancer.

[53] Beau-Faller M (2013). Rare EGFR exon 18 and exon 20 mutations in non-small-cell lung cancer on 10 117 patients: a multicentre observational study by the French ERMETIC-IFCT network. Ann. Oncol..

[54] Yu HA, Riely GJ (2013). Second generation epidermal growth factor receptor tyrosine kinase inhibitors in lung. Cancers J. Natl Compr. Canc. Netw..

[55] Sequist LV (2010). Neratinib, an irreversible pan-ErbB receptor tyrosine kinase inhibitor: results of a phase II trial in patients with advanced non-small-cell lung cancer. J. Clin. Oncol..

[56] Wu YL (2017). Dacomitinib versus gefitinib as first-line treatment for patients with EGFR-mutation-positive non-small-cell lung cancer (ARCHER 1050): a randomised, open-label, phase 3 trial. Lancet Oncol..

[57] Sequist LV (2013). Phase III study of afatinib or cisplatin plus pemetrexed in patients with metastatic lung adenocarcinoma with EGFR mutations. J. Clin. Oncol..

[58] Wu YL (2014). Afatinib versus cisplatin plus gemcitabine for first-line treatment of Asian patients with advanced non-small-cell lung cancer harbouring EGFR mutations (LUX-Lung 6): An open-label, randomised phase 3 trial. Lancet Oncol..

[59] Yang JCH (2015). Clinical activity of afatinib in patients with advanced non-small-cell lung cancer harbouring uncommon EGFR mutations: a combined post-hoc analysis of LUX-Lung 2, LUX-Lung 3, and LUX-Lung 6. Lancet Oncol..

[60] Kosaka T (2017). Response heterogeneity of EGFR and HER2 exon 20 insertions to covalent EGFR and HER2 inhibitors. Cancer Res..

[61] Jänne PA (2011). Phase I dose-escalation study of the pan-HER inhibitor, PF299804, in patients with advanced malignant solid tumors. Clin. Cancer Res..

[62] Jänne PA (2014). Dacomitinib as first-line treatment in patients with clinically or molecularly selected advanced non-small-cell lung cancer: a multicentre, open-label, phase 2 trial. Lancet Oncol..

[63] Sequist LV (2015). Rociletinib in *EGFR* -mutated non–small-cell lung cancer. N. Engl. J. Med..

[64] Cross DA (2014). AZD9291, an irreversible EGFR TKI, overcomes T790M-mediated resistance to EGFR inhibitors in lung cancer. Cancer Discov..

[65] Jänne PA (2015). AZD9291 in EGFR inhibitor–resistant non–small-cell lung cancer. N. Engl. J. Med..

[66] Soria JC (2018). Osimertinib in untreated EGFR-mutated advanced non–small-cell lung cancer. N. Engl. J. Med..

[67] Hirano T (2015). *In vitro* modeling to determine mutation specificity of EGFR tyrosine kinase inhibitors against clinically relevant *EGFR* mutants in non-small-cell lung cancer. Oncotarget.

[68] Yang M (2016). NSCLC harboring EGFR exon-20 insertions after the regulatory C-helix of kinase domain responds poorly to known EGFR inhibitors. Int. J. Cancer.

[69] Floc’h N (2018). Anti-tumor activity of osimertinib, an irreversible mutant-selective EGFR tyrosine kinase inhibitor, in NSCLC harboring EGFR Exon 20 Insertions. Mol. Cancer Ther. Molcanther.

[70] Kim E (2013). Metabolite identification of a new tyrosine kinase inhibitor, HM781-36B, and a pharmacokinetic study by liquid chromatography/tandem mass spectrometry. Rapid Commun. Mass Spectrom..

[71] Cha MY (2012). Antitumor activity of HM781-36B, a highly effective pan-HER inhibitor in erlotinib-resistant NSCLC and other EGFR-dependent cancer models. Int. J. Cancer.

[72] Thress KS (2015). Acquired EGFR C797S mutation mediates resistance to AZD9291 in non-small cell lung cancer harboring EGFR T790M. Nat. Med..

[73] Spectrum Pharmaceuticals, Inc. *Press Releases*. https://www.sppirx.com/release.html?id=21686 (2018).

[74] Butler LM, Ferraldeschi R, Armstrong HK, Centenera MM, Workman P (2015). Maximizing the therapeutic potential of HSP90 inhibitors. Mol. Cancer Res..

[75] Kamal A (2003). A high-affinity conformation of Hsp90 confers tumour selectivity on Hsp90 inhibitors. Nature.

[76] Harrison PT, Huang PH (2018). Exploiting vulnerabilities in cancer signalling networks to combat targeted therapy resistance. Essays Biochem..

[77] Jorge, S. E. et al. EGFR Exon 20 insertion mutations display sensitivity to hsp90 inhibition in preclinical models and lung adenocarcinomas. *Clin. Cancer Res*. 10.1158/1078-0432.CCR-18-1541 (2018). 10.1158/1078-0432.CCR-18-1541 PMC6295229 30154228

[78] Piotrowska, Z. et al. Activity of the Hsp90 inhibitor luminespib among non-small cell lung cancers harboring EGFR Exon 20 insertions. *Ann. Oncol.*10.1093/annonc/mdy336 (2018). 10.1093/annonc/mdy336 30351341

[79] Tsigelny IF (2015). Molecular determinants of drug-specific sensitivity for epidermal growth factor receptor (EGFR) exon 19 and 20 mutants in non-small cell lung cancer. Oncotarget.

[80] Brand TM, Iida M, Wheeler L (2011). Molecular mechanisms of resistance to the EGFR monoclonal antibody cetuximab. Cancer Biol. Ther..

[81] Wheler, J. J. et al. Combining erlotinib and cetuximab is associated with activity in patients with non-small cell lung cancer (including squamous cell carcinomas) and wild-type EGFR or resistant mutations. *Mol. Cancer Ther*. 10.1158/1535-7163.MCT-12-1208 (2013). 10.1158/1535-7163.MCT-12-1208 PMC4138057 23963360

[82] van Veggel B (2018). Afatinib and cetuximab in four patients with EGFR Exon 20 insertion–positive advanced NSCLC. J. Thorac. Oncol..

[83] Hunter FW, Wouters BG, Wilson WR (2016). Hypoxia-activated prodrugs: Paths forward in the era of personalised medicine. Br. J. Cancer.

[84] Salem A (2018). Targeting hypoxia to improve non–small cell lung cancer outcome. J. Natl Cancer Inst..

[85] Minakata K (2012). Hypoxia induces gefitinib resistance in non-small-cell lung cancer with both mutant and wild-type epidermal growth factor receptors. Cancer Sci..

[86] Murakami A (2014). Hypoxia increases gefitinib-resistant lung cancer stem cells through the activation of insulin-like growth factor 1 receptor. PLoS One.

[87] Lu Y, Liu Y, Oeck S, Glazer PM (2018). Hypoxia promotes resistance to EGFR inhibition in NSCLC cells via the histone demethylases, LSD1 and PLU-1. Mol. Cancer Res..

[88] Liu SV (2016). Phase 2 study of tarloxotinib bromide (TRLX) in patients (pts) with EGFR-Mutant, T790M-Negative NSCLC progressing on an EGFR TKI. J. Clin. Oncol..

[89] Estrada-Bernal A (2018). Abstract A157: Antitumor activity of tarloxotinib, a hypoxia-activated EGFR TKI, in patient-derived lung cancer cell lines harboring EGFR exon 20 insertions. EGFR/Her2.

[90] Luo YH, Chen YM (2014). Recent advances in the development of mutant-selective EGFR inhibitors for non-small cell lung cancer patients with EGFR-TKI resistance. Transl. Lung Cancer Res..

[91] Gonzalvez F (2016). Abstract 2644: AP32788, a potent, selective inhibitor of EGFR and HER2 oncogenic mutants, including exon 20 insertions, in preclinical models. Cancer Res..

[92] Doebele, R. C. et al. First report of safety, PK, and preliminary antitumor activity of the oral EGFR/HER2 exon 20 inhibitor TAK-788 (AP32788) in non–small cell lung cancer (NSCLC). *J. Clin. Oncol*. **36**, 15 (suppl), 9015–9015 (2018).

[93] Hasako S (2018). TAS6417, a novel EGFR inhibitor targeting exon 20 insertion mutations. Mol. Cancer Ther..

[94] Jang J (2018). Discovery of a highly potent and broadly effective EGFR and HER2 exon 20 insertion mutant inhibitor. Angew. Chem. Int..

[95] Walter AO (2013). Discovery of a mutant-selective covalent inhibitor of EGFR that overcomes T790M mediated resistance in NSCLC. Cancer Discov..

[96] Piotrowska Z (2017). OA 12.02 final results of a phase 2 study of the hsp90 Inhibitor Luminespib (AUY922) in NSCLC patients harboring EGFR Exon 20 insertions. J. Thorac. Oncol..

[97] Vyse S, Howitt A, Huang PH (2017). Exploiting synthetic lethality and network biology to overcome EGFR. J. Mol. Biol..

[98] Forbes SA (2017). COSMIC: somatic cancer genetics at high-resolution. Nucleic Acids Res..

